# Editorial: Sex differences in sport performance

**DOI:** 10.3389/fspor.2023.1230330

**Published:** 2023-06-14

**Authors:** Franck Brocherie, François Billaut, Louise Deldicque

**Affiliations:** ^1^ Laboratory Sport, Expertise and Performance (EA 7370), French Institute of Sport, Paris, France; ^2^ Département de kinésiologie, Faculté de médecine, Université Laval, Québec, QC, Canada; ^3^ Institute of Neuroscience, UCLouvain, Louvain-la-Neuve, Belgium

**Keywords:** female athlete, women, gender bias, sexual dimorphism, exercise performance

**Editorial on the Research Topic** Sex differences in sport performance

## Introduction

The participation of women in sports at all levels, from recreational to elite, and in all structures, including national and international sport organizations (1), has notably increased with a concurrent rise in professionalism and profile of female athletes [e.g., in rugby codes; (2)]. Despite this, studies involving women remain largely underrepresented or underpowered in the field of sports science and medicine (3–6). Therefore, continuous efforts to investigate sex differences, defined by genetic, biological and physiological characteristics, and/or gender bias, defined by social, cultural, and psychological traits (7), in athletic populations is paramount in expanding our scientific knowledge and unlocking sex/gender-related evidence-based applications. This Research Topic was specifically devoted to highlight sex differences in sport training, performance and recovery across a range of sport disciplines, from the individual- to team-sport athletes. Our call intended to be multidisciplinary and focused on physical, positional, technical, tactical, psychological, nutritional and pedagogical explorations.

## The contribution

Although the number of contribution is relatively modest, it is interesting to note that the 2 brief research reports and 2 original researches included in this Research Topic have investigated elite or world-class female and male athletes who, by nature, are not easily accessible for traditional research (5).

The purpose of the first study (Torvik et al.) was to document pole and ski lengths among Norwegian elite male (*n* = 87) and female (*n* = 36) cross-country skiers in the classical and skating styles, to investigate sex differences in body-height-normalized pole and ski lengths, and to correlate these equipment variables with skiing performance within both sexes. The findings about sex and performance-level differences in body-height-normalized pole lengths are useful for technical development and regulation (e.g., double-poling vs. diagonal stride) by the International Ski Federation (FIS), as well as for the ski industry research and development in order to accommodate both male and female cross-country skiers. The second study (Yoshida et al.) investigated the kinematic characteristics of spin movements in competitive ballroom dancing performed by a world champion couple, especially holding posture and lower-limb movements, in comparison with 13 Japanese national-level competitive ballroom dancers as control group. Bearing in mind that dance is a mixture of physical expression and artistry to match music rhythm, this biomechanical analysis demonstrated significant differences between the world champion couple *vs*. the control dancers. Such results on posture and lower-limb movements could serve as reference to support training in competitive ballroom dancers by creating a podium performance profile, with possible consideration for competition assessment. The third study (Torres-Aguilera et al.) analyzed the predictive value on sports performance of plasma miR-106b-5p levels in Olympic- and world-level female (*n* = 7; 500-m records: 121.80 ± 3.80 s) and male Spanish kayakers (*n* = 8; 500-m records: 101.24 ± 1.02 s) at the beginning and at the end of a training macrocycle, as well as the potential underlying molecular mechanisms using an *in silico* approach. The results revealed a sex-dependent response of circulating miR-106-5p, with a negative relationship between miR-106-5p expression and exercise performance observed in male but not in female kayakers. Independently of sexes, the lower miR-106b-5p levels obtained in athletes with a higher performance could be related to a higher angiogenesis and up-regulation of GLUT4. Altogether, this highlights the need to consider the menstrual cycle in women to move toward personalized and sex-dependent practical recommendations for optimizing the performance and health of athletes. The fourth and last study (Grasdalsmoen et al.)—a companion paper from Silvertsen et al. (8)—examined the prevalence of mental health problems among male and female “elite” (subjectively self-reported) students compared to the general student population and further explored the linear *vs*. curvilinear association between weekly hours of training across all mental health indicators (simply put “is more always better?”). Contrastingly to others (9, 10), this study reported that (i) “elite” student athletes exhibit a reduced risk of mental health problems, higher quality of life, more positive affect, less loneliness and insomnia, as well as fewer alcohol problems, compared to the general student population; and (ii) the hours of physical exercise training were associated with better mental health and higher life satisfaction. Importantly however, females displayed a worsening of mental health across most outcome measures with increasing training volume. This certainly calls for more well-conducted and well-powered studies to identify gender-specific patterns in the associations between the amount of exercise and mental health indicators (11).

## Perspectives

Because the lack of comparative studies on sex and/or gender is the main obstacle to robustly transfer knowledge to “real world” practice, this Research Topic intended to reduce the relative dearth of sex difference research in sports science and medicine. Acknowledging that effective evidence-based applications should be “contextualized” to female sporting environment and physiological and biomechanical peculiarities among others, efforts must be continued with further genuine and effective collaboration between academics, elite sport industry, scientific funding organizations and female athletes.
